# Book Review

**DOI:** 10.1120/jacmp.v3i4.2557

**Published:** 2002-09-01

**Authors:** Tim Paul

**Affiliations:** ^1^ Department of Radiation Omcology University of California Los Angeles 200 UCLA Medical Plaza Los Angeles CA 90095‐6951

## Abstract

**Practical Applications of Internal Dosimetry.** Wesley E. Bolch, editor. 480 pp. Price: $60.00. ISBN: 1‐930524‐09‐9.

PACS number(s): 87.52.–g

Accompanying the 2002 Health Physics Society's Summer School, “Practical Applications of Internal Dosimetry” is prefaced as a supplement to the 1994 summer school text on the topic of internal dosimetry. A welcomed supplement it is. The authors wisely waste little space dispensing with the basics of internal dosimetry that can be found in several textbooks or in the Annals of the ICRP. Instead, they focus on the limitations of the basic methods and propose actions for improvement.

The text begins with a basic refresher on the organ systems most involved in the transport and deposition of internal emitters. Then, an extensive literature survey on the complex biokinetics of inhaled, ingested, and percutaneously deposited radionuclides is presented. The authors provide an overview and circumspect analysis of the literature, rather than presenting the basic biokinetics models. A long reference list is provided, which will be needed for readers less experienced in internal dosimetry.

The meat of the book starts with the framework and basic models of estimating intake and ultimately the committed effective dose equivalent (CEDE) from internalized radionuclides. Of particular importance to the more sophisticated readers are the discussions on the approach to intake assessment and considerations of real‐world conditions, and how these deviations from reference assumptions introduce significant stochastic and systematic errors. The authors provide examples and identify these factors affecting accuracy. There are lengthy discussions of error and sensitivity analysis that include cogent explanations on the myriad of terminologies.

Considerations on the design and conduct of defensible programs are discussed, and the authors drive home the point that measurement sensitivity and accuracy should drive the dose assessment methodology. The commonly overlooked problem of inadequately sensitive methods is examined. The authors challenge some commonly held ideas and the current regulatory paradigm of retrospective dose assessment of individuals based on “Reference Man” rather than the prospective approach of protection of the work environment. Additionally, they assert that in practice, there is an under reliance of personal air and fecal sampling. They present scenarios demonstrating some inadequacies in chest counting and urinalysis for some actinides.

After the programmatic discussions, there is a brief introduction to Bayesian statistics. Then several case studies are presented using the Markov Chain Monte Carlo method to provide an exact solution to the Bayesian inference problem to determine previous intakes and their dates of occurrence.

In the final chapters, medical internal dosimetry from nuclear medicine and radionuclide therapies are addressed. A review of the MIRD system is presented first, then the discussion shifts to recent developments in the area of patient specific dosimetry calculated from biokinetic distributions observed in nuclear medicine imaging series.

The strengths of this text lie in valuable analyses, commentary, and recommendations provided by the authors. In that sense, the book does not read like a textbook at all. This book is recommended for individuals charged with operating, designing, and evaluating internal dosimetry programs.

